# Mitochondria in the signaling pathways that control longevity and health span

**DOI:** 10.1016/j.arr.2019.100940

**Published:** 2019-08-12

**Authors:** Mansour Akbari, Thomas B.L. Kirkwood, Vilhelm A. Bohr

**Affiliations:** a Center for Healthy Aging, Department of Cellular and Molecular Medicine, SUND, University of Copenhagen, 2200, Copenhagen N, Denmark; b Institute of Cell and Molecular Biosciences and Institute for Ageing, Campus for Ageing and Vitality, Newcastle University, Newcastle upon Tyne, NE4 5PL, UK; c Laboratory of Molecular Gerontology, National Institute on Aging, 251 Bayview Blvd, Baltimore, USA

**Keywords:** Aging, Mitochondria, DNA repair, Energy, Lifespan, Metabolism

## Abstract

Genetic and pharmacological intervention studies have identified evolutionarily conserved and functionally interconnected networks of cellular energy homeostasis, nutrient-sensing, and genome damage response signaling pathways, as prominent regulators of longevity and health span in various species. Mitochondria are the primary sites of ATP production and are key players in several other important cellular processes. Mitochondrial dysfunction diminishes tissue and organ functional performance and is a commonly considered feature of the aging process. Here we review the evidence that through reciprocal and multilevel functional interactions, mitochondria are implicated in the lifespan modulation function of these pathways, which altogether constitute a highly dynamic and complex system that controls the aging process. An important characteristic of these pathways is their extensive crosstalk and apparent malleability to modification by non-invasive pharmacological, dietary, and lifestyle interventions, with promising effects on lifespan and health span in animal models and potentially also in humans.

## 1. Introduction

Human life expectancy has been increasing worldwide since the nineteenth century. This is an impressive achievement, but the increasing elderly population entails major health and socioeconomic challenges in the years ahead. Meeting these challenges requires multidisciplinary approaches and increased knowledge about the underlying biochemical mechanisms of aging.

Aging is commonly described as a progressive decline in organismal function over time associated with an increasing risk of disease and death. Aging is complex, involves multiple and interconnected processes, is arguably universal, and shows similarities but also variation both in rate and in physiological traits across and within species (Jones et al., 2014; Lopez-Otin et al., 2013). In humans, longevity is influenced by heredity, as well as, diverse environmental factors such as nutrients, lifestyle, pollution, medical care, childhood stress, and socioeconomic status (Blackburn et al., 2015; Ridout et al., 2017).

At the molecular level, aging is thought to be a consequence of a lifelong accumulation of stochastic damage to tissues and cellular components such as DNA, proteins, and lipids (Kirkwood, 2005). Genetic studies in model organisms have identified signaling pathways that can influence the rate of aging and lifespan across species, with prominent features of these pathways being evolutionarily conserved and having extensive functional interactions. These pathways likely evolved early in the evolutionary history of living cells as part of an adaptation mechanism to integrate key biological processes in response to the availability of the intra- and extracellular nutrients and energy substrates. However, a key question that has long interested biologists is how evolutionary forces could have acted upon these pathways to favor an aging process that is obviously not maximally beneficial to the fitness of the individual.

Although it has sometimes been suggested that aging is directly programmed, e.g. as a mechanism to sacrifice the individual in favor of kin, careful analysis shows such arguments to be generally untenable (Kowald and Kirkwood, 2016). Instead, the evolution of aging is explained through the fact that natural selection is relatively weak in its control of gene actions in later life and by the principle of optimization of allocation of resources between, on the one hand, growth and reproduction, and on the other hand, maintenance and repair (Kirkwood and Austad, 2000). In particular, the “disposable soma theory” of aging recognizes that under pressure of natural selection in the wild, where individuals mostly die relatively young, the optimal allocation of nutrients and energy would have placed a limited priority on the long-term maintenance of soma when balanced against the more urgent priorities of growth and reproduction. Accordingly, longer-lived species, which generally have adaptations (wings, shells etc.) enabling better survival in the wild, allocate more resources on maintenance than short-lived species. The significance of this theory is that it provides a direct connection between evolutionary understanding of why aging occurs with the extensive evidence for how aging is regulated through the signaling pathways that sense nutrient and cellular energy availability to control cell metabolism and energy production accordingly. In line with this view, the lifespan extension effects of dietary restriction and of pharmacological treatments including central regulators of metabolism in organisms as diverse as yeast and mice often converge on these signaling pathways one way or another.

If aging is driven by damage, the key challenges are to identify which cellular components are most likely to be implicated among the primary mechanisms underlying functional decline, and how the highly networked pathways involving damage and repair can best be dissected and understood. Mitochondria are essential for normal cellular and organismal function, and defects in the pathways that control mitochondrial DNA (mtDNA) maintenance and mitochondrial function are pathogenic (DeBalsi et al., 2017). Proper mitochondrial function and maintenance require the action of multiple mechanisms (Box 1). Subtle alterations and irregularities in these processes impair mitochondrial homeostasis and function with age (Kwong and Sohal, 2000), and are linked to age-associated functional declines such as sarcopenia (Ibebunjo et al., 2013; Marzetti et al., 2013), insulin resistance (Petersen et al., 2003), and brain aging (Mattson and Arumugam, 2018).

In this review, we discuss the highly dynamic processes that connect mitochondrial homeostasis to DNA metabolism and the conserved signaling pathways that control lifespan. We also discuss some frequently reported non-invasive life style changes and pharmacological interventions that extend lifespan and health span in a range of organisms by acting on those processes.

## 2. The apparent dichotomy of mitochondrial stress and ROS in disease and longevity

For mitochondria to respond properly to local energy need and because of their other key functions, signal transduction lines of communication have evolved between mitochondria and the nucleus called anterograde (nucleus to mitochondria) and retrograde (mitochondria to nucleus). Such signaling can be enhanced in response to nuclear DNA damage (Fang et al., 2016b), mtDNA damage (Chae et al., 2013), and mitochondrial stress, such as accumulation of misfolded proteins within mitochondria (Zhao et al., 2002). The key mediators of the mitochondrial retrograde signaling pathways include ROS, Ca^2+^, and the cellular AMP/ATP ratio.

High levels of ROS are generated in abnormal mitochondria, but also ROS produced under regular physiological conditions can generate oxidative damage to cells and tissues, and have long been considered a significant driving force in the aging process, a concept known as the “free radical theory” of aging (Harman, 1956). Emerging evidence, however, has resulted in a more nuanced view and ROS are now recognized as important signaling molecules in a number of key biological processes (Sena and Chandel, 2012), including longevity (Schulz et al., 2007). For example, while severe defects in mitochondrial function are the primary cause of a number of human diseases (Schapira, 2012), in *C. elegans* mild mitochondrial stress and modest increase in mitochondrial ROS levels seem to increase lifespan (Feng et al., 2001; Lee et al., 2003b). This is reminiscent of hormesis, the concept based on observations that exposure to low doses of a toxin might have beneficial effects for the organisms, for instance, by activating cellular defense systems against stress (Mattson, 2008). Accordingly, uncritical use of antioxidant supplementation to lower ROS levels seems to have adverse effects on health and may even be linked to increased mortality (Bjelakovic et al., 2014; Ristow et al., 2009). On the other hand, in longer living animal models, multiple studies have shown that enhanced mitochondrial antioxidant capacity can extend lifespan and health span and may even explain the exceptional longevity of the naked mole rat (Munro et al., 2019; Schriner et al., 2005; Shabalina et al., 2017). These results illustrate the challenging work of understanding the complex nature of the antioxidant system and mitochondrial ROS-mediated signaling and their role in health and aging, which are further complicated by the study design, type of model system (i.e. intact cells vs. isolated mitochondria, cell lines vs. animals, short-lived vs. long-lived animals, type of tissue studied), and the limitations of the available techniques (Kalyanaraman et al., 2012; Munro et al., 2019; Sanz, 2016).

## 3. Key nutrient and energy sensing signaling pathways that control longevity in laboratory animal models

### 3.1. The somatotropic axis

**Fig. 1.**
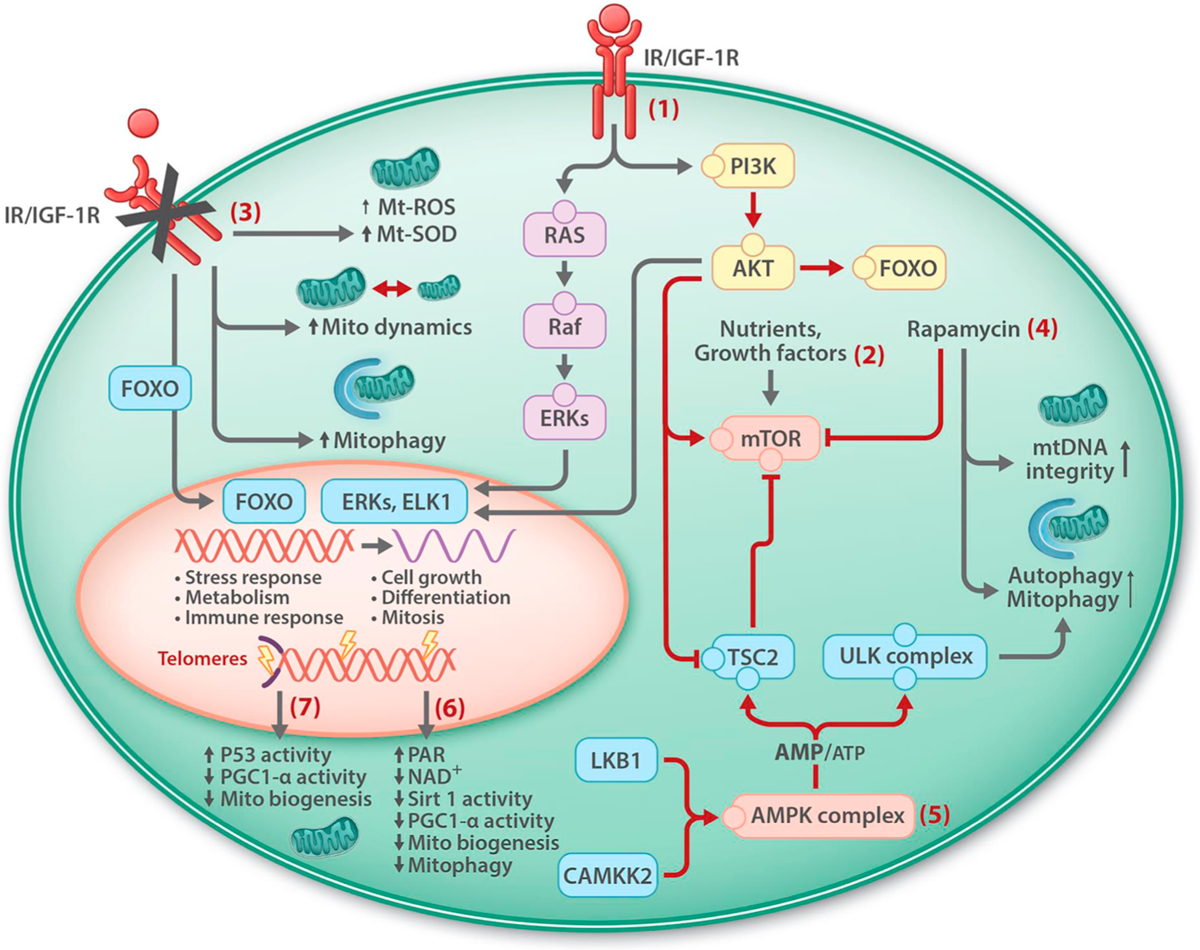
Mitochondrial homeostasis and signaling pathways that regulate longevity and health span. Signaling pathways that are activated by insulin and insulin-like growth factor 1 receptors (IR/IGF-1R) includes PI3K-Akt and Ras-Raf-ERK-MAPK pathways leading to the retention of FOXO in cytoplasm, cell growth and proliferation **(1)**, and stimulation of mTOR, and inhibition of autophagy/mitophagy **(2)**. Inhibition of IR/IGF-1R stimulates pathways that regulate mitochondrial maintenance and leads to the translocation of FOXO into the nucleus where it controls the expression of a number of genes including those involved in stress response and immunity **(3)**. Rapamycin **(4)** and activated AMPK, following increased AMP/ATP ratio **(5),** inhibit mTOR and stimulate mitochondrial maintenance. NAD^+^ over-consumption following DNA damage, and increased PARylation, reduces SIRT1 activity and mitochondrial biogenesis and maintenance **(6)**. Damage to telomeres may impair mitochondrial biogenesis through activation of p53 and reduced PGC1-a activity **(7)**.

Genomic and intervention studies in humans and other animals have identified the evolutionarily conserved somatotropic axis consisting of growth hormone (GH), insulin and insulin-like-growth factor 1(IGF-1) signaling (IIS), and their receptors and downstream effectors, as a key signaling pathway in delaying aging and in promoting longevity across species (Brown-Borg, 2015; Kenyon et al., 1993; Morris et al., 1996; Tatar et al., 2001) (Fig. 1).

GH, also called somatotropin, is a peptide hormone produced and secreted by somatotrope cells in the anterior lobe of the pituitary gland. A key function of GH is the stimulation of IGF-1 production by the hepatic cells. Circulating IGF-1 is mainly produced by liver cells, but is also produced locally in other tissues acting in paracrine and autocrine manners. IGF-1 inhibits the release of GH from the pituitary gland through a negative-feedback (Bartke et al., 2013). The amount of circulating GH and IGF-1 decline with age (Junnila et al., 2013). Individuals carrying loss of function mutation in the GH receptor are characterized by a high level of circulating GH, dwarfism and obesity, known as Laron dwarfism (Godowski et al., 1989).

Binding of insulin and IGF-1 to their receptors results in the activation of the phosphoinositide 3-kinase (PI3K), serine threonine Akt kinase, and phosphorylation of the forkhead box protein O (FOXO) transcription factor (Fernandez and Torres-Aleman, 2012) resulting in its retention in the cytoplasm. Inhibition of IGF-1 signaling results in the nuclear localization of FOXO and activation of the expression of its target genes that are involved in various cellular processes including autophagy, apoptosis, and stress responses (Lee et al., 2003a; Murphy et al., 2003). Activated Akt suppresses autophagy by stimulating mTOR and inhibiting tuberous sclerosis complex 2 (TSC2). The Ras-Raf-ERKs-MAPK pathway is another central pathway activated by insulin and IGF-1 that promotes cell growth and proliferation (Fernandez and Torres-Aleman, 2012) (Fig. 1).

#### 3.1.1. IIS pathway in aging

The influence of IIS signaling on longevity was first demonstrated in long-lived daf-2 and age-1 *C. elegans* mutants (Friedman and Johnson, 1988; Kenyon et al., 1993). The effect of daf-2 mutation on aging was suppressed in worms by an additional mutation in the daf-16 gene, revealing that DAF-2 and DAF-16 function in the same pathway (Dorman et al., 1995; Kenyon et al., 1993). Later works showed that DAF-2 is a homologue of the human insulin and IGF-1trans-membrane tyrosine kinase receptors (Kimura et al., 1997), and that AGE-1 is a worm homolog of human PI3K, and DAF-16 a human FOXO homolog acting downstream to DAF-2 and AGE-1 (Lin et al., 1997; Ogg et al., 1997). DAF-16 regulates the expression of a number genes involved in the stress-response and antioxidant defense systems, metabolism, and antimicrobial system, a sort of immune response, (Honda and Honda, 1999; Murphy et al., 2003). *C. elegans* and *D. melanogaster* have a single *FOXO* homolog gene, but mammals have four; *FOXO1*, *FOXO3*, *FOXO4*, and *FOXO6* (Martins et al., 2016).

Two closely related types of mice, the Ames dwarf mice and the Snell dwarf mice carry mutations in Prop1 (Prophet of Pit-1), a transcription factor that regulates the expression of PIT-1 that drives the differentiation of the anterior pituitary gland (Sornson et al., 1996). These mice are deficient in GH, prolactin, and thyroid-stimulating hormone (TSH), have reduced levels of IGF-1 and insulin, and increased life-expectancy.

The lifespan extension effect of diminished IIS signaling has been demonstrated in yeast (Fabrizio et al., 2001), *D. melanogaster* (Clancy et al., 2001), and mice (Brown-Borg, 2015; Holzenberger et al., 2003; Mao et al., 2018), indicating that IIS is as an evolutionarily conserved pathway integral to the aging process.

A key question is at what time-point in life targeting IIS signaling can have beneficial effects on health and lifespan. Treating 18 months old mice with an antibody against the IGF-1 receptor improved health span and increased median lifespan by 9% in female mice (Mao et al., 2018), suggesting that inhibition of IIS signaling can delay aging even when applied in advanced age, at least for females.

The role of IIS signaling in human aging is complex and not fully understood. There is, however, some evidence suggesting that reduced IIS signaling extends lifespan and health in humans as well. The GH secretion rate was found to be lower and tightly controlled in the offspring of long-lived families (van der Spoel et al., 2016). Moreover, genetic variants of the IIS signaling pathway have been identified in long-lived individuals maybe linking the IIS pathway to longevity or to a healthy life span (Milman et al., 2014; Suh et al., 2008).

#### 3.1.2. Mitochondria in IIS signaling

Biochemical and cell biological studies in animal models have identified an intricate interplay between mitochondrial physiology and IIS signaling. Inhibition of the *C. elegans* DAF-2 leads to the nuclear translocation of the DAF-16 transcription factor and the expression of the DAF-16 target genes including those involved in the regulation of cellular stress-responses and metabolism (Honda and Honda, 1999; Murphy et al., 2003). Extensive overlap was identified in the gene expression profile of daf-2 mutants and some long-lived mitochondrial mutant strains of *C. elegans* (Senchuk et al., 2018), suggesting the convergence of daf-2 and mitochondria retrograde signaling on the same lifespan controlling pathway(s). Protein synthesis is a highly energy consuming process in the cell. DAF-2 deficiency was accompanied by an overall slower protein turnover rate, which was particularly more significant for mitochondrial proteins (Dhondt et al., 2016). In a separate study, several mitochondrial parameters were markedly different in daf-2 worms such as increased abundance of OXPHOS proteins, higher reserve respiration capacity, and higher membrane potential (Brys et al., 2010). Thus, in *C. elegans*, IIS inhibition appears to influence mitochondrial bioenergetics and biogenesis by reducing the half-life of mitochondrial proteins and by enhancing the expression of the nuclear encoded mitochondrial genes.

Mitochondrial respiration progressively declines in adult *C. elegans*, but the rate of decline was slower in daf-2 mutant worms, concomitant with more mitochondrial ROS production although without detectable adverse effect on mtDNA integrity and protein oxidative damage (Brys et al., 2010). Acute inhibition of IIS in adult *C. elegans* transiently increased ROS levels, which in turn induced the expression of ROS neutralizing enzymes superoxide dismutase (SOD) and catalase followed with reduced ROS levels (Zarse et al., 2012). These results suggest that the regulation and improvement of mitochondrial function together with ROS-mediated adaptive response contribute to the life extension effect of IIS signaling impairment in *C. elegans*.

Mitochondrial shape, size and network are largely determined by the rates of fission and fusion, and alter in response to internal and external cues such as metabolic demand, and stress. Neuronal mitochondria in *C. elegans* undergo changes in density, morphology and in axonal transport frequency and distance with age (Morsci et al., 2016). In daf-2 mutants, the age-associated decline in mitochondrial morphology and density was significantly delayed and the axonal trafficking rate of mitochondria was maintained during adulthood (Morsci et al., 2016). This study demonstrated that the status of mitochondrial dynamics and distribution are closely linked to the IIS signaling and the aging process in worms.

Mitophagy is a central process in maintaining the cellular content of functional mitochondria by removing malfunctioning mitochondria. Mitophagy was markedly enhanced in daf-2 deficient *C. elegans* (Palikaras et al., 2015), and exposure to mitophagy enhancers increased lifespan in worms (Fang et al., 2017b; Ryu et al., 2016). Dct-1 is a nematode ortholog of the mammalian mitophagy receptors NIX/BNIP3L (Palikaras et al., 2015). DCT-1 impairment markedly shortened lifespan in daf-2 mutants, demonstrating a functional connection between mitophagy and the observed lifespan extension following IIS impairment in *C. elegans* (Palikaras et al., 2015).

In long-living Ames dwarf and GHR-KO mice with defects in GH/IGF-1 signaling, there was a metabolic shift to β-oxidation and significantly higher oxygen consumption rate, lower respiratory quotient (RQ), and a higher level of PGC1-α expression and more abundant OXPHOS proteins (Brown-Borg et al., 2012), connecting IIS signaling impairment in mammals to a significant shift in mitochondrial metabolism and more efficient mitochondrial biogenesis and function (Bartke and Westbrook, 2012).

Insulin signaling in adipose tissue regulates lipid and glucose metabolism. Fat-specific insulin receptor knockout mice (FIRKO) were lean and had increased lifespan (Bluher et al., 2003). The expression of genes involved in glycolysis, TCA cycle, β-oxidation, and OXPHOS proteins, were all higher in FIRKO mice with age, while they declined in control mice (Katic et al., 2007), suggesting the involvement of mitochondrial metabolism in the life extension effect of insulin signaling deprivation in the fat tissue. Disruption of the insulin receptor in adipose and muscle tissue in adult mice, however, failed to increase life expectancy (Merry et al., 2017), indicating the complex role of the mammalian IIS signaling on lifespan.

Data on mitochondrial gene expression profiles in long-lived humans with genetic variants in GH/IGF-1 pathway (Milman et al., 2014; Suh et al., 2008), and in the offspring of long-living families with reduced circulating GH hormone (van der Spoel et al., 2016), are not available and this warrants further investigation.

### 3.2. Mechanistic target of rapamycin (mTOR) signaling pathway

mTOR signaling is an evolutionarily conserved pathway that controls cell growth and metabolism in response to nutrients, growth factors, and cellular energy levels. mTOR is present in two functionally distinct complexes; mTOR complex 1 (mTORC1) and mTOR complex 2 (mTORC2) with distinct and overlapping interacting partners, and inputs and output signaling. Central to both complexes is the serine-threonine kinase mTOR, a phosphoinositide 3-kinase related protein kinase (Saxton and Sabatini, 2017).

The activity of mTORC1 is regulated in response to the availability of nutrients (amino acids, lipids, cholesterol, and glucose), growth factors (for instance insulin and insulin-like growth factors), and also the cellular energy status, i.e. AMP/ATP ratio. Activated mTORC1 promotes cell growth (accumulation of cell mass) by promoting protein, lipid and nucleotide synthesis and suppressing autophagy (Ben-Sahra et al., 2013; Ganley et al., 2009; Hosokawa et al., 2009; Peterson et al., 2011). mTORC2 controls cell growth, proliferation and cell survival by regulating lipogenesis, glucose metabolism, the actin cytoskeleton, and apoptosis (Hagiwara et al., 2012; Jacinto et al., 2004). mTOR and IIS signaling pathways functionally interact. mTORC2 phosphorylates and activates Akt (Sarbassov et al., 2005), a key protein in IIS-PI3K-Akt signaling pathway that promotes cell survival and growth in part through phosphorylation and cytoplasmic retention and inhibition of FOXO transcription factors (Fig. 1).

#### 3.2.1. mTOR in aging

Genetic and pharmacological studies have consistently shown that inhibition of mTOR increases lifespan in yeast (Kaeberlein et al., 2005), *C. elegans* (Vellai et al., 2003), *D. melanogaster* (Kapahi et al., 2004), and in mice (Harrison et al., 2009; Wu et al., 2013b), indicating that the lifespan expansion function of the mTOR pathway is a highly conserved mechanism.

Rapamycin is an antibiotic macrolide with immunosuppressive, antifungal, anticancer, and cell proliferation inhibitory properties (Yoo et al., 2017). Rapamycin binds to its intracellular receptor FK506-binding protein 12 (FKBP12). The rapamycin-FKBP12 complex inhibits mTOR by directly binding to the FRB (FKBP12-rapamycin-binding) domain of mTOR inhibiting raptor binding mTORC1 (Brown et al., 1994; Sabatini et al., 1994; Shimobayashi and Hall, 2014; Zoncu et al., 2011). Rapamycin also inhibits mTORC2 following prolonged exposure (Sarbassov et al., 2006), which may account for the observed toxicity of chronic rapamycin treatment such as glucose intolerance and insulin resistance (Lamming et al., 2012). Rapamycin has been consistently shown to prolong lifespan in various species ranging from worm to mouse (Bjedov et al., 2010; Harrison et al., 2009; Miller et al., 2011; Robida-Stubbs et al., 2012).

Key mechanisms for the increased longevity effects of mTORC1 inhibition are thought to include an overall reduction of mRNA translation and protein synthesis, and enhanced autophagy flux (Beretta et al., 1996; Kim et al., 2011b; Saxton and Sabatini, 2017; Selman et al., 2009). The serine-threonine kinase ULK1 controls the formation of autophagosoms and autophagic flux. mTORC1 actively suppresses autophagy by phosphorylating ULK1 (Kim et al., 2011b). mTORC1 also regulates autophagy in part through phosphorylation and cytoplasmic retention of transcription factor EB (TFEB), that regulates the expression of lysosomal and autophagy genes (Martina et al., 2012; Settembre et al., 2011).

mTOR signaling controls many cellular processes and functions in a highly age and tissue-specific manner. For example, mice with adipose-specific depletion of raptor had significantly less adipose tissue, did not develop obesity and showed improved insulin sensitivity and increased mitochondrial respiration in adipose tissue (Polak et al., 2008). Disruption of raptor in skeletal muscle, on the other hand, resulted in muscle dystrophy (Bentzinger et al., 2008). In a separate study, uncontrolled mTORC1 activity in skeletal muscle resulted in severe muscle atrophy because of impaired autophagy (Castets et al., 2013). These results indicate the importance of tight regulation of the mTORC1 pathway and careful implementation of mTORC1 inhibitors in longevity studies (Arriola Apelo et al., 2016; Kraig et al., 2018).

#### 3.2.2. Mitochondria in the mTOR pathway

Cell growth and proliferation are energy consuming processes, so, as could be expected, mTOR has been found to control mitochondrial function at several levels (Bao et al., 2015; Bentzinger et al., 2008; Betz et al., 2013; Cunningham et al., 2007; Khan et al., 2017; Lu et al., 2015; Morita et al., 2013; Polak et al., 2008; Ramanathan and Schreiber, 2009).

Subcellular localization of mTOR complexes is tightly linked to their function (Betz and Hall, 2013). Mitochondria-associated endoplasmic reticulum (ER) membranes (MAM) are part of ER that physically connects ER to mitochondria and are important for the movement of lipids and calcium between these organelles (Rizzuto et al., 1998). Growth factors stimulate localization of mTORC2 to MAM where it phosphorylates Akt that in turn phosphorylates MAM associated proteins IP3R and hexokinase II, thereby controlling mitochondrial physiology (Betz et al., 2013).

An important downstream function of mTORC1 signaling is the suppression of autophagy.

For example, sustained mTORC1 activity in skeletal muscle resulted in severe muscle atrophy probably as a result of impaired autophagy (Castets et al., 2013). A general increase in autophagy following mTORC1 inhibition could also be expected to enhance selective removal of damaged mitochondria by mitophagy. This is supported by several recent reports. TSC2 is a negative regulator of mTORC1 (Fig. 1). TSC2^−/−^ cells showed impaired autophagy and PINK1-mediated mitophagy (Bartolome et al., 2017). Neurons from TSC2^−/−^ mice showed impaired mitochondrial dynamics and were depleted of axonal and presynaptic mitochondria. Blocking mTORC1 restored mitochondrial homeostasis and subcellular distribution of mitochondria (Ebrahimi-Fakhari et al., 2016). In a separate study, inhibition of mTORC1 induced mitophagy in cytoplasmic hybrid (cybrid) cell lines carrying severe mtDNA defects (Gilkerson et al., 2012). Long term rapamycin treatment significantly reduced the frequency of mtDNA carrying deletion mutations in aging mice probably through stimulation of mitophagy (Bielas et al., 2018).

Leigh syndrome is a primary mitochondrial disease caused by mtDNA deletion. Treatment of a mouse model of Leigh syndrome with rapamycin improved mitochondrial function and alleviated symptoms of the disease (Johnson et al., 2013). Moreover, low dose rapamycin treatment extended lifespan in a mouse model of human mtDNA depletion syndrome probably through systemic changes in metabolism (Siegmund et al., 2017). mTOR inhibition may therefore represent a potential therapeutic target in diseases caused by defect in mtDNA maintenance systems. Collectively, these studies demonstrate that mitochondrial physiology and homeostasis are affected by the mTOR signaling pathway. We speculate that mitochondrial homeostasis is a critical mediator of the longevity effects of mTOR inhibition. Investigating this link is important, because of the key roles of mTOR signaling and mitochondrial metabolism in aging, and their malleability to regulation by non-invasive, dietary, and pharmacological interventions.

### 3.3. Adenosine monophosphate kinase (AMPK)

ATP is the principal energy substrate of the cell. The free energy released from the hydrolysis of ATP to ADP is harvested to drive almost all energetically unfavorable reactions in the cell. Therefore, elaborate systems have evolved to maintain sufficient and continual supply of cellular ATP. AMPK has been identified as the primary sensor of cellular ATP levels and a key regulator of cellular energy homeostasis and metabolism (Hardie et al., 2016; Herzig and Shaw, 2018). AMPK is a heterotrimer, evolutionarily conserved protein kinase consisting of αβγ subunits (Ross et al., 2016). Phosphorylation of Thr172 within the kinase domain of the α-subunit enhances the protein kinase activity of AMPK by several orders of magnitude. The tumor suppressor liver kinase B1 (LKB1) and the calcium-sensitive kinase (CAMKK2) are two major upstream kinases for the Thr172 phosphorylation (Hardie et al., 2016; Herzig and Shaw, 2018) (Fig. 1). AMPK senses the amount of available ATP in the form of high AMP/ATP or ADP/ATP ratios that increases Thr172 dephosphorylation and AMPK activation (Gowans et al., 2013). Activated AMPK regulates a number of metabolic processes (Hardie et al., 2016; Herzig and Shaw, 2018). In general, activated AMPK alters metabolism towards increased catabolic processes such as autophagy and down-regulates energy consuming anabolic, and biosynthetic processes including lipid and protein synthesis, to stimulate ATP production and to restore cellular energy homeostasis (Hardie et al., 2016; Herzig and Shaw, 2018).

#### 3.3.1. AMPK in aging

AMPK may influence aging and lifespan through different mechanisms (Burkewitz et al., 2014). The lifespan extension effects of pharmacological and dietary interventions in model organisms often implicate autophagy. AMPK activates autophagy by phosphorylating ULK1, a critical kinase for autophagy initiation (Egan et al., 2011), and by suppressing mTORC1 via activation of its negative regulator TSC2 (Inoki et al., 2003), and phosphorylation and inhibition of the mTORC1 subunit, raptor (Gwinn et al., 2008) (Fig. 1).

AMPK also modulates the signaling pathways that promote longevity such as those involving FOXOs (Greer et al., 2007) and SIRT1 (Canto et al., 2009).

In addition to rapamycin, multiple studies have shown that compounds such as resveratrol and metformin also increase lifespan in various organisms. AMPK is involved in mediating the life extension effects of these molecules (Burkewitz et al., 2014).

Dietary restriction (DR) is the severe reduction of food intake. DR is one of the most consistent interventions to delay the onset of age-related diseases and to increase life expectancy in organisms ranging from yeast to mammals. Long-term and acute DR activates AMPK (Burkewitz et al., 2014). Like rapamycin, a key outcome of both DR and activated AMPK is the inhibition and suppression of mTOR signaling, suggesting a DR mediated lifespan regulatory function of AMPK via inhibition of mTOR signaling pathway.

In mammals, the three AMPK subunits exists in several isoforms encoded by different genes (Ross et al., 2016). Changes in the phosphorylation and the expression of different isoforms seem to be affected by age (Salminen et al., 2016), resulting in a decline in the capacity of AMPK signaling to respond to different stimuli.

AMPK activity is suppressed by IIS via Akt (Kovacic et al., 2003), and activated AMPK inhibits IIS by phosphorylating insulin receptor substrate 1 (IRS) (Tzatsos and Tsichlis, 2007), and mTOR signaling pathways through phosphorylation of TSC2 and raptor (Gwinn et al., 2008). mTOR in turn activates IIS and is stimulated by IIS (Sarbassov et al., 2005) (Fig. 1). These examples illustrate the intricate network of interactions that regulate the longevity pathways.

#### 3.3.2. AMPK and mitochondria

The central role of AMPK in the regulation of cellular energy metabolism implies close links between AMPK activity and mitochondrial biology. Consistent with this, different mouse models of AMPK deficiency have demonstrated that AMPK influences various aspects of mitochondrial biology (Garcia-Roves et al., 2008; Lantier et al., 2014; O’Neill et al., 2011). AMPK regulates mitochondrial biogenesis by controlling the expression of mitochondrial genes. Early experiments in mice showed that a reduced ATP/AMP ratio in skeletal muscle induced the expression of PGC1-α and activated the mitochondrial biogenesis (Zong et al., 2002). This response was abrogated in mice expressing a dominant negative mutant of AMPK (Zong et al., 2002). Later work showed that phosphorylation of PGC1-α by activated AMPK enhances PGC1-α action and the induction of mitochondrial genes (Jager et al., 2007).

The dynamic changes in mitochondrial morphology and network formation by fission and fusion are integral in adaptive cell metabolism, and in the maintenance of a functional mitochondria population by mitophagy (Sebastian et al., 2017). Recent studies have implicated AMPK in an intricate signaling network that controls mitochondrial quality. An important role of AMPK in mitochondrial homeostasis is probably linked to its key function in initiating autophagy and mitophagic turnover of dysfunctional mitochondria by phosphorylation and activation of ULK1, as has been demonstrated in human cell lines and in *C. elegans* (Egan et al., 2011). Mitochondrial stress promoted mitochondrial fragmentation through phosphorylation of mitochondrial fission factor, MFF, by AMPK to facilitate elimination of damaged mitochondria by mitophagy in human and mouse embryonic fibroblasts (MEF) cells (Toyama et al., 2016). In *C. elegans*, inhibition of mitochondrial dynamics blocked AMPK- and DR-mediated increased longevity, illustrating the close interplay between mitochondrial dynamics and AMPK in regulating longevity (Weir et al., 2017). It now seems evident that mitochondrial function declines with age (Gonzalez-Freire et al., 2018). Aging-related reductions in AMPK activity and signaling may in part contribute to the reduced mitochondrial function and biogenesis over time (Reznick et al., 2007).

Altogether, evidence indicates that AMPK plays a central role in different aspects of mitochondrial homeostasis as part of an intricate signaling network with significant implications on cell metabolism and longevity.

## 4. Genome instability

Evidence indicates that DNA damage and mutation accumulate with age in multiple human and animal tissues, and loss of genome integrity, i.e. nuclear and mitochondrial DNA, is a hallmark of aging across species (Bua et al., 2006; Chow and Herrup, 2015; Fakouri et al., 2018; Fayet et al., 2002; Kalfalah et al., 2015; Li et al., 2017, 2016; Lopez-Otin et al., 2013; Mattson and Arumugam, 2018; Maynard et al., 2015; Vaidya et al., 2014; Zhang and Vijg, 2018; Zhang et al., 2015).

DNA is constantly damaged at high frequency by spontaneous hydrolytic decay and by various endogenous and environmental causes (De Bont and van Larebeke, 2004; Lindahl, 1993). DNA damage refers to any changes to the chemistry of DNA and misincorporated but chemically normal nucleotides, e.g. during DNA replication. Structural DNA damage can give rise to mutation during replication and thus change the original nucleotide sequence of DNA. While DNA damage can be repaired by the DNA repair machinery, mutations cannot, and will become fixed in the genome of the daughter cells (Akbari and Krokan, 2008). In the case of germ cells, mutations can potentially be passed on to the progeny, unless the cell dies or the carrier does not reproduce or dies at a pre-reproductive age (Crow, 1997).

Our genome is organized within the nucleus and mitochondria. The nuclear genome in a typical haploid cell contains approximately three billion base pairs and an estimated over 20 000 protein coding genes. By comparison, mtDNA is a very small ˜16.6 kb circular DNA, and contains only 13 protein coding genes. Despite its small size, however, mtDNA is crucial for development, normal cell function, and survival (El-Hattab and Scaglia, 2013). The transformation of mtDNA damage to mutation seems to occur at a much lower frequency than that of nuclear DNA (Valente et al., 2016). Thus, mutational load in nuclear and mtDNA may have distinct origins, and replication error is commonly considered the major source of mtDNA mutation (DeBalsi et al., 2017).

The preservation of the chemical structure and the original nucleotide sequence of the genome are essential for life. Thus, mechanisms have evolved in all living organisms to repair DNA lesions and to maintain genome stability and integrity (Iyama and Wilson, 2013; Sykora et al., 2012). Structure distorting lesions, such as UV-light, generate thymidine dimers that are repaired by the nucleotide excision repair (NER) pathway (Marteijn et al., 2014). DNA double strand breaks (DSBs) are repaired by non-homologous end-joining (NHEJ) (Kragelund et al., 2016), and recombination repair (San Filippo et al., 2008) pathways. Mismatch repair (MMS) is coupled with DNA replication and corrects wrongly inserted nucleotides during replication (Jiricny, 2006). DNA base excision repair (BER) pathway repairs a number of different damaged bases and non-coding baseless sites (AP sites) (Krokan and Bjørås, 2013). Genotoxic insults that generate breaks in one of the two DNA strands, called single-strand DNA breaks, often result in chemically modified 3´- and 5´-termini and are repaired by single-strand break repair (SSBR) pathway. BER and SSBR are enzymatically similar pathways and share proteins.

The expression of DNA repair proteins is up-regulated in long-lived specifies compared with animals with shorter lifespan (MacRae et al., 2015), and genetic variation in DNA repair can influence longevity (Debrabant et al., 2014; Keane et al., 2015; Kim et al., 2011a). These examples demonstrate the principle of the optimization of allocation of resources between maintenance and reproduction in the aging process.

Segmental progeroid syndromes, or premature aging syndromes, are a group of hereditary diseases that are characterized by some but not all clinical features of normal aging progressing at an accelerated rate (Carrero et al., 2016). Most cases of progeroid syndromes are causally linked to defects in DNA repair (Carrero et al., 2016; Kipling et al., 2004). Although defect in DNA repair does not seem to be a major cause of age-associated genome instability, premature aging diseases illustrate a key role of DNA repair in the aging process. Moreover, evidence suggests that the catalytic activity of DNA repair proteins alters or declines with age (Atamna et al., 2000; Cabelof et al., 2002; Xu et al., 2015), which may diminish the capacity of cells to cope with the burden of DNA damage resulting in the accumulation DNA lesions and genome instability over time. Although a number of studies on human materials and in laboratory animals have identified DNA repair as a key determinant of the rate of aging and lifespan, evidence showing positive effects of stimulation or increased DNA repair capacity on lifespan is limited and more work of this sort needs to be conducted in animal models (Qian et al., 2018).

A less investigated but potentially important source of DNA damage is the process of DNA repair itself. Alterations in the expression of DNA repair proteins during aging have been frequently reported for almost all DNA repair pathways (Gorbunova et al., 2007). Most DNA repair pathways are multi-enzyme and multistep processes and changes in the level of proteins can lead to loss of coordination in the repair process (Fu et al., 2012; Kidane et al., 2014; Senejani et al., 2012). For example, BER corrects various types of DNA lesions that occur frequently and in large numbers, which if left unrepaired can be mutagenic and cytotoxic (Krokan and Bjørås, 2013). However, uncontrolled BER activity, particularly under conditions of enhanced DNA lesions, can also lead to the generation of DNA repair intermediates, such as AP-sites and single-strand nicks, that are often highly mutagenic and cytotoxic (Akbari et al., 2009), leading to the activation DNA damage response (DDR) and cell death (Ebrahimkhani et al., 2014; Parrish et al., 2018). Thus the control and balance of all the individual steps in the DNA repair process are central to its function and changes that up-regulate or down-regulate the DNA repair processes can render the system dysfunctional.

Addition of a methyl group to cytosine (5mC) and its removal constitute epigenetic DNA modification processes that are important for the regulation of expression of a number of genes. DNA demethylation is controlled by the coordinated actions of Ten-eleven translocation (TET) proteins and BER. TET proteins oxidize 5mC to 5-hydroxymethylcytosine (5hmC) and further to 5-formylcytosine (5fC) and 5-carboxylcytosine (5caC). These bases are excised and repaired by BER resulting in the conversion of 5mC to C (Wu and Zhang, 2017). DNA methylation/demethylation processes are dynamically regulated in brain cells and continually change in response to physiological neuronal activity (Guo et al., 2011). Aberrant and incomplete BER (Weissman et al., 2007) can result in genome instability at these sites following cycles of DNA methylation/demethylation (Mahfoudhi et al., 2016), thus contributing to age-related somatic genome instability and pathological alteration of gene expression and neurodegeneration (Leandro et al., 2015).

DNA damage contributes to aging in various ways. DNA lesions can persist over a long period of time and increase with age (Siddiqui et al., 2015). Patients with childhood cancer who were treated with DNA damaging chemotherapeutic agents show signs of accelerated aging later in life (Ness et al., 2013). DNA lesions can impede transcription, which is especially highly relevant in long genes (Vermeij et al., 2016). DNA damage can affect the large number of enhancers that are located throughout the human genome perturbing normal gene expression (Andersson et al., 2014; Vijg et al., 2017). Increased amounts of DNA lesions can result in aberrant cell cycle re-entry in post-mitotic neurons leading to apoptosis or senescence (Fielder et al., 2017), and cause stem cell exhaustion (Nijnik et al., 2007; Rossi et al., 2007). DNA lesions such as bulky adducts, hydrolytic deamination of bases and AP-sites can also become converted to mutations by various mechanisms (Helleday et al., 2014). Thus, accumulation of DNA lesions combined with alterations and decline in DNA repair capacity, contribute to genome instability and constitute a critical driving force in the aging process.

## 5. The mitochondrial genome in aging

Progressive alterations in the primary structure of the genomic DNA (nuclear and mitochondrial DNA) with age can compromise mitochondrial function in various ways. Mitochondria contain ˜1200 proteins (Calvo et al., 2016), only 13 of which are encoded by mtDNA and all are components of the OXPHOS system, and are essential for mitochondrial function (DeBalsi et al., 2017).

A number of pathogenic mtDNA rearrangements and single nucleotide mutations have been reported over the years (Elliott et al., 2008; http://www.mitomap.org/). A mixture of normal and mutated mtDNA molecules in a cell is known as heteroplasmy. The proportion of mutant to normal mtDNA affects mitochondrial function and determines the severity and the progression of the disease and tissue dysfunction (Schon et al., 2012). mtDNA is exclusively maternally inherited, and diseases caused by mtDNA mutation display a maternal mode of inheritance (Schon et al., 2012).

The amount of mtDNA mutations increases in multiple tissues with age (Bua et al., 2006; Corral-Debrinski et al., 1992; Fayet et al., 2002; Greaves et al., 2014; Kauppila et al., 2017; Kennedy et al., 2013; Kraytsberg et al., 2006), mostly originating from clonal expansion of mtDNA replication errors during development and spontaneous deamination of cytosine and adenine (Fayet et al., 2002; Kennedy et al., 2013). A direct link between mtDNA instability with aging phenotypes was demonstrated in mice engineered to express a proof-reading deficient variant of mitochondrial DNA polymerase γ (Kujoth et al., 2005; Trifunovic et al., 2004). These mice accumulate mtDNA mutations at large numbers and show aging features such as weight loss, alopecia, osteoporosis, and kyphosis (Kujoth et al., 2005; Trifunovic et al., 2004).

mtDNA is organized into structures called nucleoids in close association with mitochondrial inner membrane (Brown et al., 2011). Because the mitochondrial inner membrane is a major site of mitochondrial ROS production, oxidative lesions are expected to occur frequently in mtDNA (Cadenas and Davies, 2000). Current data, however, does not support a substantial contribution of oxidative mtDNA damage to age-related increase in somatic mtDNA mutations (Halsne et al., 2012; Kauppila et al., 2018; Kennedy et al., 2013).

As discussed in Box1, of several DNA repair activities that have been reported in mammalian mitochondria, BER is likely the most active DNA repair pathway in human mitochondria (Sykora et al., 2012). Alterations in mitochondrial BER activity and protein levels with age have been identified in different tissues and organisms (Garreau-Balandier et al., 2014; Gredilla et al., 2010; Szczesny et al., 2003). These changes may lower mtDNA repair capacity resulting in persistent mtDNA damage (Canugovi et al., 2014), and create DNA repair imbalance leading to the generation of genotoxic DNA repair intermediates and compromise mtDNA integrity (Harrison et al., 2005).

A somewhat overlooked consequence of mtDNA damage on aging is the effect of DNA lesions on mtDNA transcription fidelity. In addition to oxidative damage to mtDNA from ROS, environmental chemicals and endogenously produced aldehydes can generate different forms of mtDNA crosslinks and bulky adducts (Cline, 2012; Valente et al., 2016). Such DNA lesions can stop the progression of mitochondrial RNA polymerase (POLRMT) resulting in premature termination of the transcript, or the damage can be bypassed by the polymerase but result in the production of mutated transcripts (Cline, 2012; Cline et al., 2010; Sultana et al., 2017). In the nucleus, defects in transcription-coupled DNA damage repair cause Cockayne syndrome, a disease characterized by severe neurological deficiencies and accelerated aging phenotypes (Karikkineth et al., 2017), and impaired mitochondrial homeostasis (Scheibye-Knudsen et al., 2012). Thus, mtDNA transcription failure in the form of mutations and truncations can, in principle, contribute to mitochondrial dysfunction and age-associated decline in tissue function, and warrants investigation.

Damaged deoxyribonucleotide triphosphates (dNTPs) are another important source of DNA damage and mutation. Spontaneous deamination of dCTP generates dUTP that can be readily incorporated into DNA opposite adenine. Although U:A is not mutagenic, high levels of uracil incorporation followed by removal of uracil from DNA by uracil-DNA glycosylase (UNG) generate potentially mutagenic and cytotoxic AP-sites (Krokan and Bjørås, 2013). Deoxyuridine triphosphate nucleotidhydrolase (dUTPase) hydrolyses dUTP to dUMP and pyrophosphate. dUTPase is present in both nucleus and mitochondria (Ladner and Caradonna, 1997).

MTH1 protein hydrolyzes oxidized purine nucleotides such as 8-oxo-dGTP and 8-oxo-dATP to monophosphates. MTH1 is located in the cytoplasm, mitochondria and the nucleus (Kang et al., 1995). A very low amount of oxidized guanine was able to reduce the fidelity of the mitochondrial DNA polymerase γ resulting in the pre-mutagenic A:8-oxoG mismatches in DNA (Pursell et al., 2008). Moreover, primary neurons from mice deficient for Mth1 and DNA glycosylase Ogg1 (removes the oxidative base damage 8-oxoG from DNA) contained high levels of 8-oxoG in mtDNA, exhibited poor neurite outgrowth, and mitochondrial dysfunction (Leon et al., 2016). Thus, mitochondrial nucleotide pool sanitation enzymes play key roles in the preservation of mtDNA integrity, and exploring their role in the age-associated increased mtDNA damage has not been adequately addressed and merits investigation.

Ribonucleotides are frequently mis-incorporated into genomic DNA (Williams et al., 2016). Ribonucleotides in nuclear DNA can cause DNA replication stress and small and large scale genome instability (Williams et al., 2016). The presence of ribonucleotides in mtDNA has long been known (Grossman et al., 1973). The mitochondrial DNA polymerase γ incorporates ribonucleotide into DNA with varying efficiency depending on the incoming nucleotide and the presence of a 3´-terminal ribonucleotide on a primer (Kasiviswanathan and Copeland, 2011). The frequency of ribonucleotide incorporation into mtDNA is influenced by the availability of nucleotides, and is elevated in cells from individuals with mutation in genes that regulate mitochondrial nucleotide pool, suggesting high levels of ribonucleotides in mtDNA might be pathogenic (Berglund et al., 2017). The biological consequence of ribonucleotides in mtDNA is largely unknown, but seems to decrease the speed of mtDNA replication (Forslund et al., 2018). In the context of aging, it will be interesting to investigate the rate of ribonucleotide incorporation and the level of ribonucleotides in mtDNA in different tissues with age (Pawar et al., 2018).

### 5.1. MtDNA variants

mtDNA may influence human biology and aging in subtle ways. mtDNA has a higher mutation and evolution rate than nuclear DNA, and mtDNA shows large within-population sequence variability (Elliott et al., 2008; Wallace, 2015). Non-deleterious, beneficial mutations that improved adaptation to environmental changes, such as climate and food, and infectious diseases, were fixed in mtDNA giving rise to different lineages or haplotypes. Further nucleotide sequence changes in the haplotypes generated clusters of related mtDNA within populations known as haplogroups (Wallace, 2015). Haplogroups may provide protection or influence the severity and susceptibility to various diseases (Carelli et al., 2006; Wallace, 2018). There are, however, also reports that do not support such associations (Goncalves et al., 2018; Hudson et al., 2012).

The maternal inheritance of mtDNA is predicted to result in a selection asymmetry and increased mtDNA mutation load and reduced fitness in males (Wolff and Gemmell, 2013). Analysis of data from a female founder lineage over several generations revealed that an mtDNA variant had a negative effect on male fitness. Thus, the pre-dominant maternal transmission of mtDNA has probably contributed to the reduction of male lifespan (Milot et al., 2017).

Experiments on laboratory models and domestic animals have shown associations between specific mtDNA variants and various phenotypic traits, as well as, functional interactions with the nuclear genome (Aw et al., 2018; St John and Tsai, 2018; Vivian et al., 2017). A transgenic mouse harboring nuclear DNA from one strain and homoplasmic mtDNA haplotype from another, revealed substantial influence of mtDNA haplotype on longevity and health span (Latorre-Pellicer et al., 2016).

These results suggest that mtDNA variants may have subtle effects on health and aging. Further, experimental studies using different animal models may provide better insight into functional interactions between mtDNA haplotypes and the longevity signaling pathways.

### 5.2. mtDNA and inflammation

The induction of acute inflammation is an integral part of the innate immune response to defend body against pathogens, bacterial and viral infections, tissue damage and the following healing process. On the other hand, non-infectious, low-grade, chronic inflammation is a major risk factor for the development of many age-associated diseases and frailty (Franceschi et al., 2017; Lopez-Otin et al., 2013; Mattson and Arumugam, 2018).

The inflammatory response is triggered by the release of mediators such as cytokines and chemokines by tissue-resident leukocytes and other cells (Medzhitov, 2008). Non-infectious endogenous sources of induction of inflammation includes cellular DNA molecules outside mitochondria and the nucleus, e.g. in the cytoplasm. Such DNA could be sensed as pathogen-associated molecular patterns (PAMPs) and damage-associated molecular patterns (DAMPs), by specific pattern recognition receptors (PRRs) and activation of immune response (Medzhitov, 2008; Netea et al., 2017).

The cyclic guanosine monophosphate-adenosine monophosphate synthase (cGAS) and stimulator of interferon genes (STING) signaling has been identified as a key sensor of cytoplasmic DNA (Ishikawa and Barber, 2008; Li and Chen, 2018; Wu et al., 2013a). cGAS binds to double-stranded DNA, independent of nucleotide sequence, as a result, cytosolic self-DNA (e.g. DNA released into the cytosol following nuclear DNA damage) and foreign DNA activate cGAS-STING signaling that results in the production of different inflammatory interferon and cytokines (Li and Chen, 2018; Wu et al., 2013a). Defects in the proteins that prevent the accumulation of cytosolic host DNA have been linked to Aicardi-Goutières syndrome (AGS) (Coquel et al., 2018; Crow et al., 2006; Rice et al., 2015; Yang et al., 2007), a rare systemic inflammatory autoimmune disease clinically characterized by early-onset encephalopathy, basal ganglia calcification, cerebral white matter abnormalities, and elevated level of type I interferon in serum and cerebral spinal fluid (Goutieres et al., 1998).

The release of mtDNA into cytoplasm, e.g. as a result of mitochondrial stress and damage, can activate cGAS-STING inflammatory response (West et al., 2015; White et al., 2014). Importantly, removal of damaged or stressed mitochondria by mitophagy inhibited this response (Nakahira et al., 2011; Sliter et al., 2018). Mitochondria play various pivotal roles in the regulation of innate and adaptive immune responses (Weinberg et al., 2015). Thus, the health beneficial effects of life style and compounds that stimulate mitophagy and maintain mitochondrial quality (Fang et al., 2019, 2016a; Fang et al., 2017a) are likely in part also to control chronic inflammation, a major source of age-related frailty and morbidity.

## 6. From nuclear DNA damage response to mitochondria dysfunction and aging

As we discussed in section 4, damage to DNA can cause a number of unpredictable adverse effects on protein coding genes and transcriptional regulatory regions. However, recent findings that DNA lesions (nuclear and telomeric) generate signaling responses that influence mitochondrial function (Fakouri et al., 2018; Fang et al., 2016b; Sahin et al., 2011), have identified a novel consequence of an overall effect of stochastic genomic damage on cell function and aging, thus connecting genome instability to mitochondrial dysfunction, two hallmarks of aging (Lopez-Otin et al., 2013; Mattson and Arumugam, 2018).

Poly (ADP-ribose) polymerases 1 and 2 (PARP1/2) are activated following binding to DNA damage to signal the site of damage to DNA repair proteins by adding ADP-ribose polymers (PAR) to themselves and to the nearby proteins while consuming nicotinamide adenine dinucleotide (NAD^+^) in the process (Pascal and Ellenberger, 2015).

NAD^+^ is an important redox coenzyme in metabolic pathways such as the citric acid cycle, and glycolysis, and is also used as co-substrate by three classes of enzymes; 1- poly (ADP-ribose) polymerases (PARP1/2), 2- sirtuins, and 3- the cyclic ADP-ribose (cADPR) synthases (CD38, CD157) (Canto et al., 2015). These enzymes compete for the available cellular NAD^+^ pool. The level of NAD^+^ declines with age (Braidy et al., 2011; Zhu et al., 2015), and in animal models of human DNA repair deficiency (Fang et al., 2016a, 2014), while NAD^+^ supplementation improves health span and extends lifespan in different animal models (Mouchiroud et al., 2013; Zhang et al., 2016).

Sirtuins are evolutionarily conserved protein deacetylases/deacylases implicated in the regulation of aging and longevity in various organisms (Imai and Guarente, 2016). Seven sirtuins (1–7) have been identified in human cells that localize in the nucleus (SIRT1, 6 and 7), cytoplasm (SIRT2) and in mitochondria (SIRT 3–5) (Canto et al., 2015). The nuclear SIRT1 controls and regulates transcription by deacetylating transcription factors, and modulates the catalytic activity of enzymes (Canto et al., 2015).

**Fig. 2.**
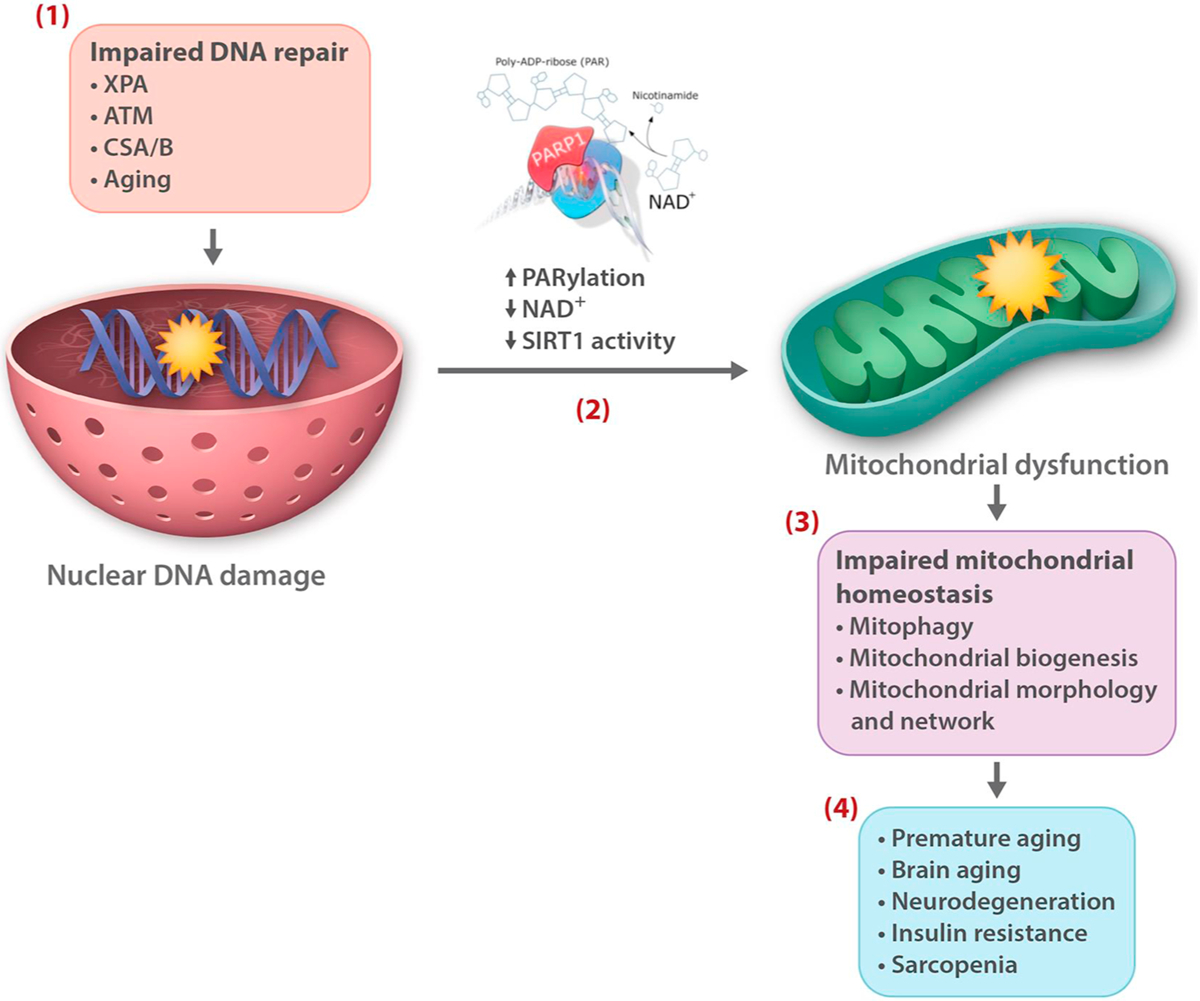
NAD^+^ over-consumption following persistent DNA lesions is a central link between nuclear DNA instability and mitochondrial dysfunction, two hallmarks of aging. Defects in DNA repair proteins such as in the premature aging disorders xeroderma pigmentosum group A (XPA), ataxia telangiectasia mutated (ATM), and Cockayne syndrome A and B (CSA/B), as well as age-related DNA repair imbalance and dysregulation (Aging), result in the accumulation of DNA damage and genome instability **(1)**, leading to prolonged PARP activation (PARylation), increased NAD^+^ consumption, and reduced NAD^+^ levels and SIRT 1 activity **(2)**. A consequence of this chain of events is impaired mitochondrial homeostasis, e.g, because of reduced autophagic turnover of mitochondria by mitophagy and mitochondrial biogenesis **(3)**, that in DNA repair deficient disorders contribute to premature aging phenotypes, and in normal aging process leads to diminished tissue and organ function and disease **(4)**.

Several recent studies have identified a link between nuclear DNA damage and cell metabolism and mitochondrial homeostasis through NAD^+^ consumption. For example, increased PARP activity, as a result of prolonged genotoxic exposure, defects in DNA repair, and during the aging process, limits the level of NAD^+^ available for sirtuins consequently affecting the function of their downstream targets such as PGC1-α, an important regulator of mitochondrial biogenesis and proteins involved in mitophagy (Bai et al., 2011; Fang et al., 2019). Accordingly, NAD^+^ supplementation and PARP inhibition increases cellular NAD^+^ levels and improves mitochondria homeostasis in various animal models of human diseases (Fang et al., 2019, 2016a; Fang et al., 2017a) (Fig. 2).The effect of DNA damage-mediated PARP activation on mitochondrial bioenergetics and cell metabolism is complex, probably involving alternative pathways (Fakouri et al., 2018; Fang et al., 2017a; Fouquerel et al., 2014), and requires more investigation.

## 7. Telomeres

The regular DNA replication machinery is not able to synthesize the ends of the chromosomes. The maintenance and the protection of the genes at the end of the chromosomes is achieved by telomeres, the tandem repeats of TTAGGG nucleotide sequences, and telomerase, a specialized reverse transcriptase, that replicates and maintains telomeres. Telomeres are partially protected from damage by a protein complex called shelterin that consists of TRF1, TRF2, POT1, RAP1, TIN2, and TPP1 subunits (de Lange, 2005).

In many human cell types, telomeres shorten throughout the life-span; however, a critical role of telomere shortening in the usual human aging process is not fully established. Most studies connecting telomere attrition to aging phenotypes have been conducted in transgenic animal models with defects in telomere maintenance genes, in which telomere shortening during normal aging is insignificant (Blackburn et al., 2015).

Cell senescence is probably a key link between telomere attrition and aging. Many cell types express telomerase at a very low level. When these cells are cultured, they undergo progressive telomere shortening during successive rounds of cell division and eventually enter a permanent cell cycle arrest known as replicative senescence. Telomeres are particularly vulnerable to oxidative damage that can result in telomere shortening and dysfunction and persistent DDR (Barnes et al., 2019; Hewitt et al., 2012). It has been speculated that the higher sensitivity of telomeres to oxidative damage was to help telomere-driven replicative senescence to block the growth of the most badly stressed cells that were at high risk of accumulating DNA damage and mutations (von Zglinicki, 2002).

Damage to telomeres can induce senescence independently of telomere size by activating DDR (Anderson et al., 2019; Hewitt et al., 2012).

### 7.1. Mitochondria and telomeres

Reciprocal signaling between telomere damage and mitochondrial function has been reported and appears to occur through different mechanisms. ROS emitted from dysfunctional mitochondria was identified as a major determinant of telomere damage and telomere dependent senescence, which was proposed to account for cell-to-cell variation in replicative capacity (Passos et al., 2007).

In telomerase deficient mice, persistent telomere damage and attrition was shown to activate the transcription factor p53, which in turn suppressed the expression of PGC1-α and PGC-1β genes that are considered key regulators of mitochondrial biogenesis, thus compromising mitochondria homeostasis (Sahin et al., 2011). Telomere dysfunction and p53 activation also suppressed the expression of sirtuins including the mitochondrial sirtuins 3–5, which was corrected by p53 deletion (Amano et al., 2019). Administration of the NAD^+^ precursor NMN, lowered the level of p53 acetylation (which can negatively affect p53 transcriptional activity), maintained telomere length, and reduced DDR. In addition, NMN treatment improved several mitochondrial parameters in transgenic mice (Amano et al., 2019). The results of this study identify a novel pathway linking impaired sirtuin activity following telomere dysfunction and DDR, to mitochondrial dysfunction, and present NAD^+^ supplementation as a potential therapeutic approach for telomere-related aging phenotypes and diseases.

It should, however, be noted that patients with dyskeratosis congenita, a telomere related disease caused, in part, by mutation in the components of telomerase complex, do not seem to show clear clinical features of mitochondrial disease, (Armanios and Blackburn, 2012; Scheibye-Knudsen et al., 2013), but this needs further investigation.

Other forms of apparent interplay between mitochondria and telomeres involve the dual localization of TERT, the catalytic subunit of telomerase, and the shelterin protein TIN2 in mitochondria and telomeres.

A number of studies have demonstrated the presence of TERT in mitochondria both in proliferating and post-mitotic brain neurons. However, whereas initial studies showed that localization of TERT in mitochondria compromised mitochondrial function and mtDNA integrity (Santos et al., 2004, 2006), later works demonstrated improved mitochondrial parameters and mtDNA stability (Ahmed et al., 2008; Haendeler et al., 2009).

Compared to TERT, limited data is available on the biological significance of mitochondrial localization of TIN2. Overexpression of TIN2 had a profound effect on mitochondrial morphology and network. Furthermore, shRNA mediated TIN2 knockdown improved several parameters of mitochondrial bioenergetics, (Chen et al., 2012), suggesting a link between TIN2 and mitochondrial morphology and metabolism.

The RECQL4 helicase functions in DNA repair and replication and is a central enzyme in the maintenance of genome stability (Croteau et al., 2014). RECQL4 localizes into mitochondria and contributes to telomeric DNA maintenance (Croteau et al., 2014, 2012; Ghosh et al., 2012). Individuals with defect in RECQL4 develop premature aging syndromes which may be in part related to its role in telomere integrity and mtDNA metabolism.

In addition, a number of DNA repair proteins have functions in the mitochondria and in telomeres (Akbari et al., 2014; Batenburg et al., 2012; Duxin et al., 2009; Safdar et al., 2016; Sahin et al., 2011; Aamann et al., 2010). It is not clear what drives the distribution of these proteins between mitochondria and telomeres, and this presents as an interesting avenue of future research.

Taken together, there appears to be some crosstalk between mitochondria and telomeres, and elucidating functional links between these compartments merits more investigation.

## 8. Mitochondria in cellular senescence

Cellular senescence has long been recognized as a distinct and central feature of aging tissues (Dimri et al., 1995). It is characterized by a state of irreversible and permanent proliferation arrest in somatic cells, together with a complex and distinct senescence-associated secretory phenotype (SASP) consisting of upregulation of pro-inflammatory cytokines, proteases, and growth and angiogenesis factors that can alter tissue homeostasis (Freund et al., 2010; Wiley and Campisi, 2016). Senescence can be induced by various stress factors including mitogenic stress, and persistent DNA damage including telomeric DNA, and activation of DDR, and is commonly thought to have been developed to counter oncogenic transformation of the affected cell (Wiley and Campisi, 2016). Over time, the number of senescent cells increase, and this results in diminished tissue function, altered tissue structure, and decline in tissue repair and regenerative capacity, collectively promoting aging and age-related phenotypes (Chapman et al., 2019). Accordingly, genetic and pharmacological clearance of senescent cells delays the onset of age-related pathologies and extends health span in mice (Baker et al., 2011; Xu et al., 2018).

Evidence suggests a key role for mitochondria in the development of cellular senescence and in the modulation of the SASP signaling. A range of mitochondrial alterations have been reported in senescent cells including mitochondrial morphology and network, mass, mitochondrial ROS production, metabolites, and overall function (Chapman et al., 2019; Kaplon et al., 2013; Moiseeva et al., 2009; Wiley and Campisi, 2016; Ziegler et al., 2015).

Controlled disruption of mitochondrial function and depletion of mitochondria in cells, have provided some clues about the molecular processes that connect mitochondrial metabolism to senescence.

Cells treated with various DDR activators and senescence inducers display increased mitochondrial mass prior to senescent cell cycle arrest (Moiseeva et al., 2009). This is via transcriptional activation of PGC1-β resulting in increased mitochondrial ROS production and DNA damage. Persistent DNA damage, gives rise to a positive feedback loop through ATM, Akt, and mTORC1 phosphorylation activation followed by enhanced PGC-1β dependent mitochondrial biogenesis, sustained DDR, cell cycle arrest and cellular senescence (Correia-Melo et al., 2016).

Mitochondrial dysfunction-associated senescence (MiDAS) is characterized by distinct SASP, and reduced NAD^+^/NADH ratio and AMPK activation. Activated AMPK was shown to drive growth arrest and senescence though phosphorylation of p53 that increased expression of its target senescence markers p21^WAF1^ and p16^INK4a^ (Wiley et al., 2016). Senescence and MiDAS phenotypes were also detected in tissues from transgenic mice that accumulate mtDNA mutations, and dysfunctional mitochondria, and show accelerated aging phenotypes (Kujoth et al., 2005; Trifunovic et al., 2004), demonstrating MiDAS in vivo (Wiley et al., 2016).

Thus, cellular senescence might be an important consequence of age-related decline in mitochondrial homeostasis. Because of the key roles of senescence and mitochondrial dysfunction in aging and age-related tissue dysfunction and disease, more in vivo studies in animal models and in human tissues are needed.

Collectively, current data suggests that cellular senescence integrates several regulators of longevity and health span, i.e. genome instability and DDR, mitochondrial metabolism, AMPK activation, and mTOR pathway function, which are largely druggable targets, including senescent cells and SASP (Arriola Apelo et al., 2016; Bai et al., 2011; Fang et al., 2016a, a; Kirkland et al., 2017; Kraig et al., 2018; Lin and Hardie, 2018; Zhang et al., 2016), which can also be modified by lifestyle changes (Di Francesco et al., 2018; Mattson et al., 2018).

## 9. Dynamic modeling of modulators of longevity and health span

**Fig. 3.**
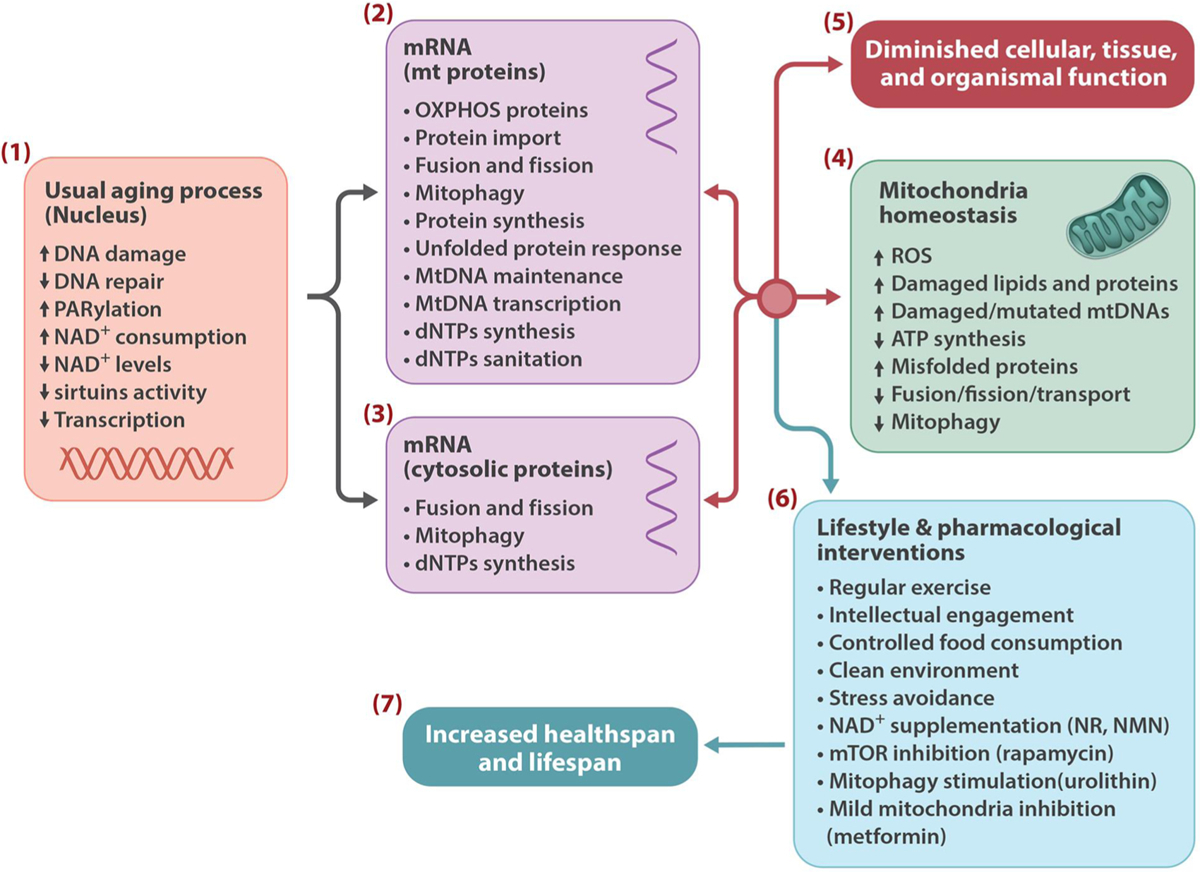
Multiple processes may link nuclear DNA instability to mitochondrial dysfunction and aging, several of which are malleable to modification by lifestyle and drug interventions. Persistent DNA damage and DNA damage response (PARylation), reduces cellular NAD^+^ content resulting in aberrant activity of sirtuins and their downstream targets **(1)**. These events can affect mitochondrial proteins **(2)** and cytosolic proteins that control mitochondrial quality and mtDNA maintenance **(3)**, leading to mitochondrial stress and loss of mitochondrial homeostasis **(4)**, which ultimately result in tissue and organismal dysfunction **(5)**. Life style and pharmacological interventions **(6)** may improve health span and increase lifespan by correcting these events **(7)**.

A central objective in aging research is to develop intervention strategies to reduce age-associated illness and frailty, and to improve quality of life at old age. The action of the longevity signaling pathways is influenced by highly dynamic and complex system of interactions and feedback loops, and is controlled at several levels including gene expression, subcellular localization, and post-translational modification, and importantly by external factors like lifestyle and various compounds (Figs. 1–3).

Computer modeling has been developed to handle complex biological systems (Dalle Pezze et al., 2014; Guimera et al., 2019; Mc Auley et al., 2017). Integrated dynamic computer modeling represents a powerful tool to bring together the actions and responses determined experimentally in order to quantify and predict the outcomes of various drug interventions on the longevity regulating pathways and mitochondrial function, and to develop testable models for intervention strategies.

## 10. Conclusions

The biology of the aging process is complex, shows large inter-individual variations, and is largely driven by the accumulation of stochastic damage to tissues and cellular components over time.

Impaired mitochondrial function and homeostasis is now commonly recognized to constitute a central connection to multiple aspects of the aging process and age-onset frailty and diseases (Lopez-Otin et al., 2013; Mattson and Arumugam, 2018). This is largely because of the central position of mitochondria in key cellular processes, as well as, the control of mitochondrial homeostasis by numerous processes taking place within and outside the mitochondria. Interestingly, mitochondrial dysfunction has emerged as a malleable therapeutic target (Fang et al., 2019, 2016a; Ryu et al., 2016). This seems to be a research area of great opportunity going forward.

Single cell and multicellular organisms have evolved interconnected signaling mechanisms to sense and respond to genome damage, and to the environmental cues and nutrients to regulate their internal needs for growth, proliferation and maintenance. Research has consistently shown that the manipulation of some of these pathways markedly affects lifespan and health span in various model organisms. A remarkable outcome of these studies is the identification of lifestyle and pharmacological interventions that act on these pathways and can potentially delay or reduce age-associated chronic illnesses and extend lifespan (Fang et al., 2017a, b; Mattson et al., 2018) (Fig. 3). Some of the drugs that promote health span and extend lifespan in animal models are already in clinical use for other purposes, and some seem safe to be tested in human clinical trials (Dellinger et al., 2017; Martens et al., 2018; Newman et al., 2016).

With the advancing knowledge and the growing insight into the genetic and biochemistry of aging, together with continual improvement in biomarkers of biological aging, safer and target specific intervention strategies may be developed to improve mitochondrial biology and to modulate the longevity signaling pathways to provide a healthier aging for the growing elderly populations worldwide.

## Box 1 The intricate mechanisms of maintaining mitochondrial function and homeostasis.

Mitochondria are the primary sites for the production of cellular ATP, and also play a central role in several other key cellular processes such as apoptosis (Gahl et al., 2016), cytoplasmic calcium buffering (Drago et al., 2012), and reactive oxygen species (ROS) mediated signaling pathways (Shadel and Horvath, 2015). The dual location of mitochondrial genes, the dynamic nature of mitochondria, the bidirectional mitochondria-nucleus signaling, together with the mechanisms for the degradation of dysfunctional mitochondria, form an intricate and interconnected network important for normal mitochondrial and cellular functions.

Each mitochondrion contains multiple copies of ˜16.6 kb circular mtDNA molecules. mtDNA contains 37 genes that code for two ribosomal RNAs (rRNAs), 22 transfer RNAs (tRNAs), and 13 proteins. mtDNA is crucial for organismal development, normal function, and survival.

The mechanism of mtDNA replication is complex, and several models have been proposed (Holt and Reyes, 2012). The enzymes critical for mtDNA replication (mtDNA replisome) *in vitro*, are the heterodimer mtDNA polymerase γ, DNA helicase Twinkle, mitochondrial single-strand DNA-binding protein (mtSSB), mitochondrial RNA polymerase (POLRMT), topoisomerase 3α and DNA ligase III (Holt and Reyes, 2012; Nicholls et al., 2018).

mtDNA is organized into structures called nucleoids in close association with the mitochondrial inner membrane (Brown et al., 2011). Because the mitochondrial inner membrane is a major site of mitochondrial ROS production, (Cadenas and Davies, 2000) mtDNA is expected to constantly undergo oxidative damage. DNA base excision repair (BER) is the prominent DNA repair pathway for repair of oxidative DNA damage, and likely to be the most active DNA repair pathway in human mitochondria (Sykora et al., 2012). Other DNA repair activities in mammalian mitochondria have been reported, including mismatch repair (de Souza-Pinto et al., 2009), and double-strand break repair (Bacman et al., 2009; Tadi et al., 2016).

mtDNA repair and replication require a steady supply of nucleotides that are recycling inside the mitochondria, as well as those imported from the cytoplasm. Defects in genes that regulate the supply of mitochondrial nucleotides cause mtDNA depletion syndromes (MDS) characterized by severe reduction in mtDNA content and impaired mitochondrial function that usually affect one or several organs (El-Hattab and Scaglia, 2013).

Mitochondria form interconnected networks, and adapt cell-type specific morphology and undergo fusion and fission (Giedt et al., 2016). The major factors in the fusion process include mitofusin 1(MFN1), mitofusin 2 (MFN2), and optic atrophy type 1 (OPA1). The central fission proteins include the dynamin-related protein 1 (DRP1), mitochondrial fission factor (MFF), mitochondrial fission 1 protein (Fis1), and mitochondrial dynamic proteins MiD49, and MiD51 (Chan, 2012; Loson et al., 2013). Mutations in *MFN2* and *OPA1* cause neurological disorders, Charcot-Marie-Tooth disease (Zuchner et al., 2004), and Autosomal Dominant Hereditary Atrophy (ADOA) (Delettre et al., 2000), respectively. Diminished expression of OPA1 has been reported in cells from ataxia with oculomotor apraxia type 1 (AOA1) patients (Zheng et al., 2019).

Autophagy is the mechanism for targeting cytoplasmic material from endogenous and exogenous sources to lysosomes for degradation, such as protein aggregates and organelles (Galluzzi et al., 2017). Selective autophagic elimination of mitochondria is called mitophagy. Mitophagy is an important control system for the removal of damaged mitochondria (Rodger et al., 2018), during development and differentiation (Schweers et al., 2007), and other conditions (Al Rawi et al., 2011; Fang et al., 2019). PTEN-induced putative kinase 1 (PINK1) and Parkin, a cytosolic E3 ligase, are key factors in mediating mitophagy. Mutations in PINK1 and Parkin are linked to the hereditary form of Parkinson disease (Kitada et al., 1998; Valente et al., 2004). A number of factors and receptors for the initiation and formation of mitophagy cargo (mitophagosome) have been identified (Rodger et al., 2018).

The processes of fusion, fission, and mitophagy are tightly connected and collectively determine mitochondrial morphology and dynamics (Sebastian et al., 2017).

## Nomenclature

AMPK: Adenosine monophosphate kinase
ATM: Ataxia telangiectasia mutated
ATP: Adenosine 5´-triphosphate
BER DNA: base excision repair
*C. elegans*: Caenorhabditis elegans
DDR: DNA damage response
DR: Dietary restriction
*D. melanogaster*: Drosophila melanogaster
ETC: Mitochondrial electron transport chain
ELK: ETS-Like 1 protein
ERK1/2: Extracellular signal-regulated kinase 1/2
FOXO: Forkhead box O
GH: Growth hormone
IIS: Insulin and insulin-like-growth factor (IGF-1) signaling
MAPK: Mitogen-activated protein kinase
mtDNA: Mitochondrial DNA
mTOR: Mechanistic target of rapamycin
NAD^+^: Nicotinamide adenine dinucleotide (oxidized)
NMN: Nicotinamide mononucleotide
OXPHOS: Oxidative phosphorylation
PGC1-α: Peroxisome proliferator-activated receptor-gamma co-activator-1α
ROS: Reactive oxygen species
SOD: Superoxide dismutase
TCA cycle: Tricaboxylic acid cycle
